# A hidden gem on the banks of the Danube River—our experience at the 28^th^ Budapest Nephrology School

**DOI:** 10.1080/0886022X.2025.2453010

**Published:** 2025-02-02

**Authors:** Joshua H. Lipschutz, Mihály Tapolyai

**Affiliations:** ^a^ Department of Medicine, Medical University of South Carolina, Charleston, SC, USA; ^b^ Department of Medicine, Ralph H. Johnson Department of Veterans Affairs Medical Center, Charleston, SC, USA; ^c^ Department of Medicine, Szt. Margit Hospital, a Semmelweis University Teaching Hospital, Budapest, Hungary

**Keywords:** Education, nephrology, transplantation, Europe, medicine

Along the banks of the Danube, I found a hidden gem. I was invited to be a Faculty member for the ‘28^th^ Budapest Nephrology School (Nephrology, Hypertension, Dialysis, Transplantation, Nephropathology)’ organized since 1994 by the Hungarian Kidney Foundation and its founding president, Dr. László Rosivall, under the auspices of the International Society of Nephrology (ISN) and the European Renal Association (ERA) with assistance from Drs. Orsolya Cseprekál and Nóra Ledó, all of Semmelweis University (https://bns-hungary.hu/). Semmelweis University named after the great Dr. Ignaz Semmelweis who, despite intense resistance from the medical community who were offended by his suggestions that they were somehow unclean, proved that hand washing with chlorinated solutions dramatically reduced infection and mortality associated with childbirth [1]. This conference, located in a series of stately old-world buildings in downtown Budapest brings together students and faculty from around the world, building on a strong tradition of learning for almost three decades [2].

The conference began on Monday, 8/26/24, with an outstanding talk on the genetic basis of hypertension along with interesting clinical cases by Dr. Freidrich Luft of Germany who is a veteran of twenty-five of these conferences! On a personal note, Dr. Luft was my professor when I was a medical student and resident at Indiana University and was one of the main reasons I went into Nephrology so it was great to reconnect with him. His talks were followed by four lectures by Dr. Jens Titze of Duke and the National University of Singapore who discussed cutting-edge research on the role of salt in blood pressure and adrenal hormone response as well as the mechanism of SGLT2 inhibitors. We also heard about the development of nucleoside-modified mRNA lipid nanoparticle vaccines and lipid nanoparticle mRNA carriers and vaccine adjuvants by Dr. Norbert Pardi from the University of Pennsylvania (Penn), where I was faculty for thirteen years. This was followed by dinner and the ‘8th Renal Wine Competition’ with special dishes and selected quality wines from the best-known Hungarian cellars at the Domus Vinorum Wine house.

Tuesday brought talks by Dr. Janos Peti-Peterdi of the University of Southern California on intravital imaging of glomerular pathobiology and new mechanisms in kidney tissue regeneration. These were followed by a talk on the kidney-brain axis and cognitive impairment in CKD by Dr. Georgina Gyarmati also of the University of Southern California. Dr. Katalin Susztak, another Penn faculty member who is originally from Hungary, then gave three talks on her pioneering research in diabetic nephropathy, genetics in chronic kidney disease, and APOL1 in chronic kidney disease. The lectures were rounded out by Kitty Jager of the Amsterdam University Medical Centers who spoke about the art (and joy!) of scientific writing and bias and confounding in scientific papers. We finished the day with a visit to the original historical building of the Semmelweis University followed by a cruise on the Blue Danube.

On Wednesday, Dr. Peter Studinger of Semmelweis University gave us the latest recommendations for treatment of renovascular disease. This was followed by an autosomal dominant polycystic kidney disease (ADPKD) session by Dr. Albert Ong of the University of Sheffield in the U.K. who discussed the genetics, diagnosis, prognosis, treatment and management of ADPKD. I then talked about our work with the exocyst complex and its role in ciliogenesis, cystogenesis, and PKD as well as a possible novel treatment, β2-adrenergic receptor agonists, that we have identified to treat diabetic nephropathy. The sessions ended early so that we could all go on a sightseeing tour of Budapest which included a visit to the ornate Parliament building.

Thursday began with a transplantation session including more talks from Penn Faculty. Dr. Simin Goral from Penn spoke about HBV, HCV, HIV in kidney transplantation with the latest data on transplants from HCV- and even HIV-positive donors. She then showed the latest data on kidney transplantations and pregnancy followed by a talk on the management of recipients with a failed kidney transplant. Dr. Ondrej Viklicky of the Institute of Clinical and Experimental Medicine (IKEM) in Prague spoke about how to overcome ABO and HLA barriers in living donor kidney transplantation along with the molecular assessment of kidney allografts and HLA incompatible transplantation. Dr. Eva Honsova of Prague finished the day by discussing renal amyloidosis and a practical approach to the diagnosis of intratubular casts. A farewell dinner was enjoyed that evening at the Urbán Betyár restaurant.

On Friday, Dr. Attila Mócsai from Semmelweis presented their latest findings on mouse models of glomerulonephritis, then Dr. András Tislér from Semmelweis spoke about highlights in clinical nephrology over the past year. In the afternoon, Dr. Safak Mirioglu of Istanbul University talked about the ERA and the Young Nephrologists Platform (YNP). This was followed by research talks from the Hungarian YNP. Dr. Ádám Hosszú of Semmelweis discussed his research on the Sigma-1 receptor, a novel drug target for renal disease. Dr. Gergő Attila Molnár of the University of Pécs in Hungary talked about nephrology, diabetes, and hypertension with a focus on oxidative stress and modified amino acids. Dr. László Rosivall then gave concluding remarks, highlighting the unfading importance of person-to-person education and adjourned the Budapest School of Nephrology…at least until next year.

If you are looking to spend some time next August in a great city while learning about the latest in Nephrology from experts around the world, I can only recommend the 29^th^ Budapest School of Nephrology.

Joshua H. Lipschutz M.D., F.A.S.N.

Professor of Medicine and Renal Division Director,

Medical University of South Carolina

Senior Clinician Scientist Investigator

Ralph H. Johnson Department of Veterans Affairs Medical Center

Charleston, South Carolina U.S.A.

**A view from inside Hungary-Comments from the Journal’s Deputy Editor:**

Had it not been disrupted by the COVID pandemic, Budapest Nephrology School (BNS) could have organized its 30th anniversary in August 2024. Very well displayed during the 28th BNS, this initiative of Professor László Rosivall is based on pathophysiology, evidence-based medicine and the deeply rooted nephrology in the history and traditions of Hungarian medical/clinical practice. Although during some part of the past century a decline in Hungarian nephrology seemed inevitable, a new wave of its revitalization came up in the late 80s and early 90s. Indeed, the establishment of the Hungarian Kidney Foundation by Professor Rosivall in 1987, as the first foundation in Hungary after the WWII, was a humble but important beginning for tireless and dedicated efforts over about four decades not only to bring back Hungarian nephrology to its deserved international levels, but also provide a platform for regional improvements in the Central and Eastern Europe. BNS as one of the major activities of the Hungarian Kidney Foundation has undertaken this task and therefore it is rightfully supported by ISN and ERA. This task has been recognized and supported by giants of international nephrology such as Eberhard Ritz, Tom Andreoli, Gerald Schulman who was instructed by Roscoe Robinson, Simin Goral, Andrzej Wiencek, Jens Titze, among others. Many others started as students in BNS, then collaborators and are now among internationally recognized nephrology researchers, such as Janos Peti-Peterdi, Istvan Mucsi and Miklos Molnar, achieving academic prominence in North America, just to name a few.

In 28 years, Budapest Nephrology School has created an academic and nurturing environment for the young physicians and nephrologists from around the world to meet and learn with the best international experts of nephrology, transplantation, and dialysis from molecules to bedside care. BNS provides scholarships and financial assistance to those doctors who cannot otherwise afford attending such international events. Courses are European and United States CME credited and the BNS collaborates closely with others, having established a sister-institution relationship with both Vanderbilt University Medical School and with the University of Toronto. Some participants reflected that they felt extremely privileged to spend a week, in close encounter and communication, with the ‘Best of the Best’ in nephrology. They found themselves in a situation, similar to none other, where they can interact with the professors to learn with them and take home invaluable messages both in academia, clinics, and personal human experience which can never be transferred in an official conference or an online course. Budapest has recently been selected and introduced as the best location for tourism; maybe we can similarly introduce this city as the best location for a successful nephrology training course.

Semmelweis University gets its name from a well-known and self-less medical doctor known as the Savior of Mothers, Ignac Semmelweis [3]. However, this university was also the home for scientists such as Alexander von Koranyi (1866-1944), who was a world-class academician, researcher and clinician in nephrology [4]. He was the first who coined the term “renal insufficiency” and defined it at the molecular level. For the first time, he measured the osmotic activity of plasma and urine to deduct the concentrating capacity of the kidneys [5]. He created a School of Thought for his students who later continued his path of exploration. Hungary has been traditionally strong in nephrology research and practice. BNS has set another example in this School of Thought and has left remaining impressions in the lives of many young medical doctors.

Despite the fact that education is becoming more and more dependent on technologies such as online and artificial intelligence, we should not underscore enough the value and importance of face-to-face, interactive and humane education. BNS continues to provide a platform, organized by humans, to facilitate cultural and scientific interaction between learners and educators in a week-long course full of memorable experiences that cannot be earned in any other environment. We look forward to further recognition and development of the Budapest Nephrology School in the coming years and expanding its’ attraction beyond Europe and North America to the Far East and Africa, going forward.

Mihály Tapolyai, M.D., Ph.D., F.A.S.N., F.A.C.P.

Senior Consultant Nephrologist Szt. Margit Hospital, a Semmelweis University Teaching Hospital, Budapest, Hungary

Staff Nephrologist, Ralph H. Johnson Department of Veterans Affairs Medical Center, Charleston, South Carolina U.S.A.
